# Identification of Orthosteric GABA_B_ Receptor Ligands by Virtual Screening and *In Vitro* Validation

**DOI:** 10.1021/acsomega.5c02102

**Published:** 2025-05-16

**Authors:** Linn S. M. Evenseth, Clizia Russotto, Imin Wushur, Dawid Warszycki, Angel S. Moldes-Anaya, Andrzej J. Bojarski, Mari Gabrielsen, Ingebrigt Sylte

**Affiliations:** † Pharmacology and Toxicology, Department of Medical Biology, Faculty of Health Sciences, 60482 UiT The Arctic University of Norway, NO-9037 Tromsø, Norway; ‡ Department of Medicinal Chemistry, Maj Institute of Pharmacology, 69714 Polish Academy of Sciences, 31-342 Kraków, Poland; § Cyclotron and Radiochemistry Unit, Section for Radiopharmaceutical Production, The PET Imaging Center, 60519 University Hospital of North Norway (UNN), NO-9019 Tromsø, Norway; ∥ Center for Research and Education, University Hospital of North Norway (UNN), NO-9038 Tromsø, Norway

## Abstract

The GABA_B_ receptor (GABA_B_-R) is a heterodimeric class C G-protein coupled receptor (GPCR) associated with numerous neurological and neuropsychiatric disorders and is an interesting target for drug development. Each subunit has an extracellular part called the Venus flytrap domain (VFT), and the VFT of the GABA_B1a/b_ subunit contains the orthosteric γ-aminobutyric acid (GABA) binding site. In the present study, we have used a combined ligand- and structure-based virtual screening (VS) campaign to identify putative compounds binding to the orthosteric binding site. Based on the VS, 34 ligands were purchased and tested *in vitro* using the functional Hit Hunter cAMP assay in Chinese hamster ovary (CHO)-K1 cells stably overexpressing the human GABA_B(1b,2)_-R and in wild-type CHO-K1 cells. Based on the initial testing, two compounds were selected for studies in the [^35^S]­GTPγS binding assays and a competition binding assay using the GABA_B_-R antagonist [^3^H]­CGP54626 as the radioligand. In addition, their effects on the dose–response curve of GABA were further evaluated in the Hit Hunter cAMP assay. The experimental testing confirmed that both compounds bind to the orthosteric site of GABA_B_-R and are antagonists.

## Graphical abstract

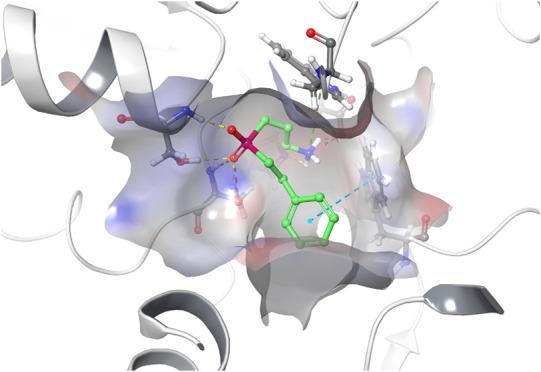

## Introduction

γ-Aminobutyric acid (GABA) is the main inhibitory neurotransmitter in the mammalian central nervous system (CNS). In the synaptic cleft, GABA functions by binding to the ionotropic GABA_A_- and the metabotropic GABA_B_ receptor (GABA_B_-R). The ionotropic GABA_A_ receptor is a ligand-gated ion channel,^1^ while the GABA_B_-R is a class C G-protein coupled receptor.^2^ The class C GPCRs consist of 23 members, including receptors for the main excitatory neurotransmitter glutamate (mGlu receptors), calcium-sensing receptor (CaSR), pheromone receptors, taste 1 receptors, and orphan receptors.^3–5^ A characteristic feature of class C is the existence of homo- or heterodimers, which is obligatory for optimal function.^3^ Each monomer contains three unique structural elements consisting of an extracellular Venus flytrap domain (VFT), which binds orthosteric agonists, a 7-transmembrane (7TM) domain, and a linker peptide domain linking the VFT and 7TM domain.^6,7^

In contrast to other class C GPCRs that are homodimers, the GABA_B_-R is an obligate heterodimer comprised of GABA_B1a/b_ and GABA_B2_ subunits.^7,8^ There are multiple isoforms of the GABA_B1_ subunit, but the most abundant are the GABA_B1a_ and GABA_B1b_, encoded by the same gene.^9^ The peptide domain linking the VFT and 7TM domains is shorter in sequence than those in other class C GPCRs and lacks the cysteine residues that are conserved among other class C members. Contrary to other class C members, only the VFT of the GABA_B1a/b_ subunit holds an orthosteric binding site.^10^ The three-dimensional (3D) structures of the GABA_B1a/b_ VFT in complex with different agonists and antagonists and of the apo form have been known for several years.^2,11,12^ These structures show that VFTs have a bilobed architecture with two distinct domains, LB1 and LB2. Binding of an agonist brings the LB1 and LB2 into closer proximity (active and closed state of the VFT) and induces a series of conformational changes that propagate a signal to the TM domain of the GABA_B2_ subunit that activates the G-protein.^13^ Binding of an antagonist prevents LB1 and LB2 from coming into close contact and stabilizes an inactive and open conformational state of the VFT.

Previous experimental and theoretical studies have suggested that an allosteric binding site is located within the 7TM of the GABA_B2_ subunit at a position corresponding to the orthosteric site in class A GPCRs.^14–16^ During the last years, several cryogenic electron microscopy (cryo-EM) structures of the full-length GABA_B_-R in apo form, as well as in both active and inactive conformations, complexed with agonists, antagonists, allosteric modulators, and G-protein have been published.^17–21^ Surprisingly, these structures show an allosteric binding site located at the interface of the 7TM domains of GABA_B1a/b_ and GABA_B2_ close to the intracellular end of the two helical bundles. Furthermore, a region within the TM domain of GABA_B2_, corresponding to the sodium binding site in class A, was found important for the activity of positive allosteric modulators (PAMs) and for controlling the constitutive activity of the receptor.^22^ The available structures show the presence of a phospholipid within the 7TM domain of the GABA_B2_ subunit at a site corresponding to the previously predicted allosteric site that may have an allosteric role in receptor activation.^17^

The GABA_B_-R couples to several intracellular signaling pathways and regulates synaptic transmission mediated through G_i/o_ proteins by either inhibiting presynaptic neurotransmitter release or dampening postsynaptic excitability.^23^ GABA_B_-R is considered an attractive target for drug discovery because its signaling pathways have been connected to a variety of neurological and neuropsychiatric disorders such as epilepsy, dementia, Fragile X syndrome and autism spectrum disorders, depression, anxiety, schizophrenia, memory and learning deficits, and drug and alcohol addiction and pain, in addition to gastroesophageal reflux disorder.^24–30^ However, the only FDA-approved drug targeting the GABA_B_-R is the agonist baclofen, which is used as a muscle relaxant and antispastic agent.^28,31^

The functional importance of the receptor emphasizes the need for developing selective compounds with tolerable side effects. Currently, most known agonists and antagonists are structural analogues of GABA.^8,32–36^ The high structural similarity between these compounds indicates that the conformational space and chemical diversity of the orthosteric compounds are yet to be explored. The present study aimed to identify new compounds targeting the orthosteric binding site of the GABA_B_-R by combining *in silico* virtual screening (VS) methods and *in vitro* assays.

## Materials and Methods

### Virtual Screening

#### Collection and Preparation of the Data Set for Virtual Screening

A database of approximately 8.2 million compounds was generated by downloading compounds from the ZINC 15 database (https://zinc15.docking.org).^37^ Drug-like compounds from the following seven vendors were downloaded: Maybridge, Enamine, Chemdiv, Chembridge, Vitas M, UORSY, and Specs. The compounds were prepared using Schrödinger LigPrep (LigPrep, Schrödinger, LLC, New York, NY, 2016) with an ionization state in the range of pH 7.4 ± 0.2, retaining the specified chirality.

#### ADMET Filtering

Schrödinger QikProp (QikProp, Schrödinger, LLC, New York, NY, 2016) was used to filter the database of 8.2 million compounds using an ADMET filter. The following parameters were applied to identify compounds for putative oral administration: 0–2 reactive groups, gut–blood barrier penetration of 300 nm/s, blood–brain barrier coefficient of −3.65 to −0.54, and logarithm of aqueous solubility of −9.2 to −2.1.

#### Pharmacophore Screening

In a previous study, we used the MOLPRINT 2D (M2D) method to generate fingerprints of 13 GABA_B_-R antagonists and 42 GABA_B_-R agonists (including enantiomers) before the compounds were hierarchically clustered using the Tanimoto similarity metric.^34^ The structural clustering resulted in six clusters: four containing agonists (clusters 2, 4, 5, and 6), one containing antagonists (cluster 3), and one containing both agonists and antagonists (cluster 1). The clusters were used to generate a 3D pharmacophore hypothesis for each cluster.^34^ In the present study, the six pharmacophore models were used to screen the data set after ADMET filtering using the Phase software, which is included in the Schrödinger Small-Molecular Drug Discovery suite of programs (Phase, 2017, Schrödinger Release 2017-4, Schrödinger, LLC, New York, NY). Mapping and matching with compounds in the database were performed by representing each feature of a pharmacophore as a distance vector. The vector must overlap with the distance vector of the mapped ligand in the database to be considered as a match. The compounds in the database should match all features of a pharmacophore to be considered as a hit. The retrieved output was separated into six groups based on the origin of the pharmacophore models, giving four groups of putative agonists (clusters 2, 4, 5, and 6), one group of putative antagonists (cluster 3), and one group of putative agonists and antagonists (cluster 1).

#### Docking Protocols and MM-GBSA Calculations

The compounds retrieved after the pharmacophore screening were used in structure-based VS steps that included different docking approaches and postprocessing by MM-GBSA calculations.^38^ The two available VFT X-ray crystal structures cocrystallized with agonists (PDB id: 4MS3 and 4MS4), and the six available VFT X-ray crystal structures cocrystallized with antagonists (PDB id: 4MR7, 4MR8, 4MR9, 4MS1, 4MRM, 4MQF) were preprocessed in the Schrödinger protein preparation wizard using default settings; hydrogen bonds were assigned with a PROPKA pH of 7, and restrained energy minimizations were performed on the structures.^39^ Grid maps were generated for the eight X-ray crystal structures with a van der Waals radius scaling factor of 1 and a partial cutoff of 0.25 Å. The cocrystallized ligands were selected as the centroid of the grid maps, and the grid sizes were increased by changing the inner box volume from 10 to 15 Å^3^ to ensure that larger compounds than the cocrystallized ligands could be docked. The remaining settings for grid generation were kept at default values.

The four groups of compounds retrieved by the agonist-based pharmacophores were docked into the two X-ray structures representing agonist-bound (closed and active) VFT structures, while compounds retrieved by the antagonist pharmacophore (cluster 3) were docked into six X-ray structures representing antagonist-based (open and inactive) VFT structures. Compounds retrieved by the pharmacophore model based on both agonists and antagonists (cluster 1) were docked in all eight X-ray crystal structures. Threshold values for scoring of docking were established in our previous study^34^ by docking the 13 antagonists and 42 agonists, initially used to generate the pharmacophore models, into prepared X-ray structures to calculate average scores. These calculations gave threshold values of −8 kcal/mol for scoring in agonist-based VFTs and −7.1 kcal/mol for scoring in antagonist-based VFTs, and these values were used to evaluate compounds in the present study. The docking protocol was performed using the stepwise virtual screening workflow (VSW) protocol in Glide, consisting of (1) High Throughput Virtual Screening (HTVS), (2) Standard Precision (SP), and (3) Extra Precision (XP) (gscore).^40,41^ The protocol was executed with a scaling factor of van der Waals radii for nonpolar atoms of 0.80 Å and a partial charge cutoff at 0.15 Å, with a postdocking minimization after each step^40,41^ (Glide, 2017, Schrödinger Release 2017-4, Schrödinger, LLC, New York, NY). In the first two steps (HTVS and SP), only 10% of the top-scored drug-like compounds were retained, while in the last step (XP), all compounds were retained, and three poses per compound were generated. The output from the XP step was postprocessed with Prime MM-GBSA calculation to estimate the relative free energy of binding.^38,41^

The retrieved compounds from the docking workflow were merged into two groups of potential agonists and antagonists. The output from the screening using the pharmacophore model generated from outliers (cluster 1) was duplicated and added to both sets. An in-house script was used to (1) select the highest-scored pose out of the three generated poses in the last docking step, (2) remove identical compounds (duplicates) based on SMILES, and (3) keep compounds with a glide docking score better than the calculated threshold (−7.2 kcal/mol for putative antagonists and −8 kcal/mol for putative agonists). The script also identified and reported which VFT X-ray structure the different compounds could dock into in terms of the PDB code.

#### Selection of Compounds for *In Vitro* Evaluation

The two new data sets generated by merging the outputs from docking calculations into agonist- and antagonist-based X-ray structures and removal and annotation of duplicates were clustered using the Kelley criterion^42^ and the Tanimoto similarity metrics after applying the M2D methodology for generation of fingerprints. A selection of complexes with compounds from each cluster was visually inspected before compounds were ranked, taking into consideration the XP gscore, relative binding affinity from the MM-GBSA calculation, and the number of X-ray structures the compounds were able to dock. In addition, similarities in receptor binding modes with known agonists and antagonists in the X-ray structures of VFTs^11^ were also considered. This ranking was used to select 34 compounds for *in vitro* testing.

### *In Vitro* Evaluation

#### Materials

Materials purchased from DiscoverX: cAMP Hunter CHO-K1 GABBR1+GABBR2 Gi Cell Line (Cat.# 95-0165C2), AssayComplete Revive CHO-K1Medium (Cat.# 92-0016RM2S), G-418 (Apollo Scientific, Cat.# BIG0175), puromycin (Fisher Scientific, Cat.# 12122530), AssayComplete CHO-K1 Cell Culture Kit 35 (Cat.# 92-0018G2R2), Hit hunter cAMP Assay for Small Molecules (Cat.# 90-0075SM2), and White clear bottom, tissue culture treated 384-well (Cat.# 92-0013). Materials purchased from Sigma-Aldrich: CaCl_2_ (Cat.# C7902), HEPES (Cat.# H3375), MgCl_2_·6H2O (Cat.# M9272), KCl (Cat.# 746436), NaCl (Cat.# 746398), d-(+)-Glucose (Cat.# G7021), NaOH (Cat.# 30620), GABA (Cat.# A5835), dimethyl sulfoxide (DMSO) (Cat.# 472301), Water-soluble forskolin NKH477 (Cat.# N3290), DME/F-12, 1:1 mixture (Cat.# D8900-10 × 1L), bovine serum albumin (Cat.# A7030-50G), polyethylenimine solution (Cat.# P3143-100 ML), Dulbecco′s phosphate-buffered saline without Ca^2+^ and Mg^2+^ (Cat.# D8537-500 ML), and 96-well flat-bottom microtiter plate (Cat.# M7687-100EA). Radioligand binding assay and [^35^S]­GTPγS assay related materials: [^3^H]­CGP54626 (American Radiolabeled Chemicals, Inc., 60 Ci/mmol, Cat.# ART 0175), [^35^S]­GTPγS (REVVITY NORGE AS, 1250 Ci/mmol, Cat.# NEG030H250UC), Grade GF/F glass fiber filter disk (VWR, Cat.# 513-5242 and Cat.# 516-0343), Ultima Gold XR liquid scintillation cocktail (Sigam-Aldrich, Cat.# L8411-5L), Ecolite (+) liquid scintillation cocktail (VWR, Cat.# ICNA0188247504), ChemiScreen GABA_B_-R membrane preparation (Eurofins, Cat.# 2150335), SafeSeal 5 mL PCR-PT tube (Sarstedt, Cat.# 4081521), GS 39783 (Tocris, Cat.# 2001), CGP54626 (Tocris, Cat.# 1088), Guanosine 5′-[γ-thio]­triphosphate tetralithium salt (GTPγS) (Millipore, Cat.# 371545-10MG), and Guanosine 5′-diphosphate sodium salt (GDP) (Sigma-Aldrich, G7127-10MG).

The wild-type (WT) CHO-K1 cell line was provided by the Tumor Biology research group at the Department of Medical Biology, Faculty of Health Sciences, UiT The Arctic University of Norway.

#### Preparation of Test Compounds

The 34 compounds for *in vitro* testing were purchased from MolPort (https://www.molport.com/). The smiles codes and MolPort ID of the test compounds are shown in Table S1. The test compounds were dissolved in 100% DMSO at a stock concentration of 10 mM and stored at −20 °C. Due to solubility problems, the stock concentration of compounds 5 and 25 was 5 mM. Stock compounds were subsequently serially diluted in 100% DMSO and finally in a d-glucose solution (20 mM NaOH, 5 mM d-glucose) to obtain final concentrations for testing.

#### Cell Culture

CHO-K1 cells stably expressing the human GABA_B(1b,2)_-R were cultured with DiscoverX culture media supplemented with antibiotics (streptomycin, penicillin, G-418, puromycin) or DME/F-12, 1:1 mixture supplemented with the antibiotics mentioned above. The WT CHO-K1 cells were cultured with the same culture medium provided by DiscoverX without adding antibiotics.

Cells were grown in a humidified incubator with 5% CO_2_ at 37 °C. The cells were grown to 75% confluence in T75 flasks. Prior to the experiment, the media were aspirated, and the cells were rinsed with ice-cold Ca^2+^/Mg^2+^ free PBS (Dulbecco’s phosphate-buffered saline) to reduce receptor internalization. The cells were harvested using a cell scraper and 50 mL of ice-cold PBS. After centrifugation at 300*g* for 5 min, the cell pellet was gently resuspended in 50 mL of ice-cold HBSS assay buffer (Hank’s balanced salt solution: 131.5 mM NaCl, 5 mM KCl, 1 mM MgCl_2_, 10 mM HEPES, 10 mM d-glucose, 1.3 mM CaCl_2_, pH 7.4). The cell suspension was centrifuged again for 5 min at 300*g*, the supernatant discarded, and the cell pellet resuspended in 6 mL of the ice-cold HBSS assay buffer.

#### Functional Hit Hunter cAMP Assay

##### GABA and Forskolin Preparation

A 40 mM GABA solution was prepared in HBSS buffer and subsequently serially diluted into the desired concentrations. Forskolin in a stock concentration of 5 mM was diluted in deionized water down to 30 μM. Each GABA serial dilution was mixed with 30 μM forskolin in a 1:1 ratio and used to obtain a GABA-Forskolin dose–response curve. A final concentration of 50 μM forskolin and 10 μM test compounds (5 μM for low solubility test compounds **5** and **25**) were prepared for testing on WT CHO-K1 cells. Mixtures of 30 μM forskolin, 10 μM test compound (5 μM for compounds **5** and **25**) in the presence of 27.4 nM and 740 nM GABA (corresponding to GABA EC_20_ and EC_80_ concentration, respectively) were used for testing in the GABA_B(1b,2)_-R expressing cells. After the harvested cells were mixed with the compound mixture in a 96-well flat-bottom microtiter plate (approximately 13.000 cells per well), the cells were shaken for 2 min at 700 rpm (VWR Microplate shaker, Cat.# 444-0270). The microplate was immediately incubated in a water bath at 25 °C for 25 min. After incubation, the cAMP detection solution was added according to the manufacturer’s instructions. After overnight incubation at room temperature, the samples were loaded on a 384-well microplate, and the luminescence was read with a BMG Labtech CLARIOstar Microplate Reader (BMG Labtech, Ortenberg, Germany). The effects of compounds **23** and **28** on the dose–response curve of GABA were also tested. 10 μM compounds **23** or **28** were added to varying concentrations of GABA (20 μM, 2.22 μM, 741 nM, 247 nM, 27.4 nM, 9.14 nM, 1.02 nM, 0.34 nM) in the Hit hunter cAMP assay. The results were compared to the standard GABA dose–response curve with the same varying concentrations of GABA.

#### Functional [^35^S]­GTPγS Assay

In a SafeSeal 5 mL PCR-PT tube, GABA_B_-R membrane preparation, 3 μM GDP, 100 μM of GABA, and 30 μM of compounds **23** or **28** were mixed in the binding assay buffer (20 mM HEPES, 100 mM NaCl, 1 mM CaCl_2_, 5 mM MgCl_2_, 0.1 mM EDTA, pH 7.4) in a final volume of 100 μL and incubated at 30 °C for 30 min. Then, [^35^S]­GTPγS was added to a final concentration of 0.4 nM and further incubated for 30 min at 30 °C before the reaction was stopped by quickly washing the assay mixture with 4 mL of ice-cold wash buffer (50 mM HEPES, 500 mM NaCl, 4 μM GTPγS, 0.1% BSA, pH 7.4) three times through a Millipore 1225 vacuum filtration Manifold preloaded with GF/F glass fiber disk. Prior to the filtration, the filter disk was presoaked in 0.78% polyethylenimine solution that has 5 μM GTPγS for 1 h and then washed with 50 mM HEPES, pH 7.4, 0.5% BSA. The basal level was determined in the absence of GABA. To test if compounds **23** and **28** bind to the orthosteric site, we tested the effect of 30 μM of these compounds on the receptor activation after activating the receptor with 1 μM GABA or with 1 μM GABA in combination with 1 μM or 10 μM of the PAM GS39783.

#### [^3^H]­CGP54626 Radioligand Binding Assay

In a SafeSeal 5 mL PCR-PT tube, the radioligand [^3^H]­CGP54626, GABA_B_-R membrane preparation, and 20 μM of test compound were mixed in the binding assay buffer (20 mM Tris, 118 mM NaCl, 4.7 mM KCl, 2 mM CaCl_2_, 1.2 mM KH_2_PO_4_, 1.2 mM MgSO_4_, 5 mM d-glucose, pH 7.4) in a final volume of 100 μL. The final concentration of [^3^H]­CGP54626 was 4 nM. After 1.5 h of incubation at room temperature, the assay mixture was quickly washed with 4 mL of ice-cold wash buffer (50 mM HEPES, 500 mM NaCl, 0.1% BSA, pH 7.4) three times through a Millipore 1225 vacuum filtration Manifold (Sigma-Aldrich, cat. no. XX2702550) preloaded with a GF/F glass fiber disk. Prior to filtration, the filter disk was presoaked in 0.33% polyethylenimine solution for 1 h and then washed with 50 mM HEPES, pH 7.4, 0.5% BSA. Nonspecific binding was determined in the presence of 10 μM unlabeled CGP54626.

#### Data-Handling and Statistical Analysis of *In Vitro* Results

Data-handling, curve fitting, and statistical analysis were performed with GraphPad Prism v. 9.0 (https://www.graphpad.com/updates/prism). Results are presented as a percentage of control, and values represent mean + standard error of the mean (SEM) (*n* ≥ 2, in duplicate or triplicate). Dose–response data obtained from the cAMP inhibition assay were fitted using a four-parameter logistic model from which EC_50_ values were inferred. All data were analyzed using the appropriate statistical test. For the dose–response data from the cAMP assay, a one-way analysis of variance (ANOVA) followed by Welch’s *t* test was used. For the data from the functional [^35^S]­GTPγS and [^3^H]­CGP54626 radioligand binding assays, Welch’s *t* test was performed. The level of significance was set to *p* < 0.05. For all experiments, a test *p*-value of <0.05 was regarded as statistically significant.

### Structural Similarity between Hits and Known GABA_B_-Receptor Compounds

#### Data Set for Similarity Analysis

281 GABA_B_-R compounds were downloaded from ChEMBL (https://www.ebi.ac.uk/chembl/), and 33 compounds were downloaded from IUPHAR (https://www.guidetopharmacology.org/) that have all been tested on the human GABA_B_-R (ChEMBL ID: CHEMBL2111463). After deleting duplicates and inactive compounds, four additional compounds were included in the data set: the recently discovered negative allosteric modulator (NAM), COR758 ^43^ and the putative NAM 13c,^44^ and the hit compounds **23** and **28**. The ligands were prepared with LigPrep (Force field: OPLS_2005, pH 7.4 ± 0.2, Epik) using Maestro (version 13.4, Schrödinger, LLC, New York, NY, 2022) for determining the protonation state of the compounds at physiological pH and obtained all possible tautomers. Finally, the data set containing agonists, antagonists, and allosteric modulators consisted of 122 compounds. Including tautomers, this gave 179 molecules for similarity analysis.

#### Hierarchical Clustering

Hashed binary fingerprints (MOLPRINT 2D)^45^ were generated for the data set before clustering using the cheminformatics package Canvas (version 5.4, Schrödinger, LLC, New York, NY, 2020). Hierarchical clustering was performed to group compounds with similar structures using Tanimoto similarity metrics and the average cluster linkage method. The similarity metrics were also calculated from fingerprints. The Tanimoto similarity coefficient ranges from 0 to 1, and structures are usually considered similar when their index is higher than 0.85.^46^

#### Induced Fit Docking of Compounds **23** and **28**

Since both compounds were proven to be antagonists, we used an antagonist-based X-ray structure (PDB id: 4MR7) for induced fit docking. This is also the available X-ray structure of GABA_B_-R VFTs with the best resolution (2.15 Å) and is complexed with the antagonist CGP54626. Both the two test compounds and CGP54626 contain aromatic groups, and in addition, [^3^H]­labeled CGP54626 was also used in our competition binding assay.

#### Protein Preparation and Ligand Generation

The antagonist-based X-ray structure (PDB id: 4MR7) was downloaded from PDB (Protein Data Bank) and subjected to modifications through the Protein Preparation Wizard, Schrödinger, LLC, New York, NY, 2024. Possible errors in the crystal structure, such as missing side chains or missing loops, were corrected. The protonation state was adjusted to pH 7.4 ± 0.2, and unwanted molecules were deleted. The structures of compounds **23**, **28**, and the antagonist CGP54626 were downloaded from PubChem (https://pubchem.ncbi.nlm.nih.gov). The four compounds underwent ligand preparation to convert the 2D structures into 3D structures using LigPrep (Schrödinger Release 2024-3: LigPrep, Schrödinger, LLC, New York, NY, 2024) using the OPLS4 force field. All possible states at pH 7.4 ± 0.2 were generated, as well as tautomers. Specified chiralities were retained, and at most, 32 isomers per ligand were generated.

#### Induced Fit Docking

The induced fit docking (IFD) was performed using the induced fit docking protocol (Schrödinger, LLC, New York, NY, 2024). The grid box was centered on the cocrystallized antagonist CGP54626 to ensure selection of the orthosteric binding site. During induced fit docking, the ring conformations were sampled in an energy window of 2.5 kcal/mol. Nonplanar conformations for amide bonds were penalized. The receptor and the ligand van der Waals scaling were set to 0.50 as the default. Residues within 5.0 of ligand poses were refined, and side chains were optimized. Using the extra precision (XP) docking protocol, the maximum number of poses was set to 20.

## Results

### Virtual Screening

ADMET (absorption, distribution, metabolism, excretion, toxicity) filtering was performed to retrieve drug-like compounds (Figure 1). The filtering reduced the number of compounds from approximately 8.2 to 5.3 million. The six pharmacophore models from our previous study^34^ were applied after ADMET filtering and reduced the number of compounds from 5.3 million to 686.031. The number of compounds retrieved by the different pharmacophore models was as follows: cluster 1, 486.074; cluster 2, 74.868; cluster 3, 10.998; cluster 4, 22.943; cluster 5, 29.366; cluster 6, 61.782. The pharmacophore model generated from outliers (cluster 1) consisting of agonists and antagonists retrieved most compounds, as expected. The features of this pharmacophore model were more general and expected to retrieve many false-positive compounds, as seen in the previous statistical evaluation.^34^ The compounds retrieved by pharmacophore screening (686.031 compounds) were docked into VFT X-ray structures. Compounds retrieved by agonist-based pharmacophores were docked into agonist-based VFTs, compounds retrieved by the antagonist-based pharmacophore were docked into antagonist-based VFT structures, while compounds retrieved by the pharmacophore of both agonist and antagonist (cluster 1) were docked into both agonist- and antagonist-based VFTs. Altogether 74.721 compounds scored better than the threshold values. Of these, 2761 scored highest in agonist-based VFT structures, while 71.960 scored highest in antagonist-based VFT structures.

**Figure 1:**
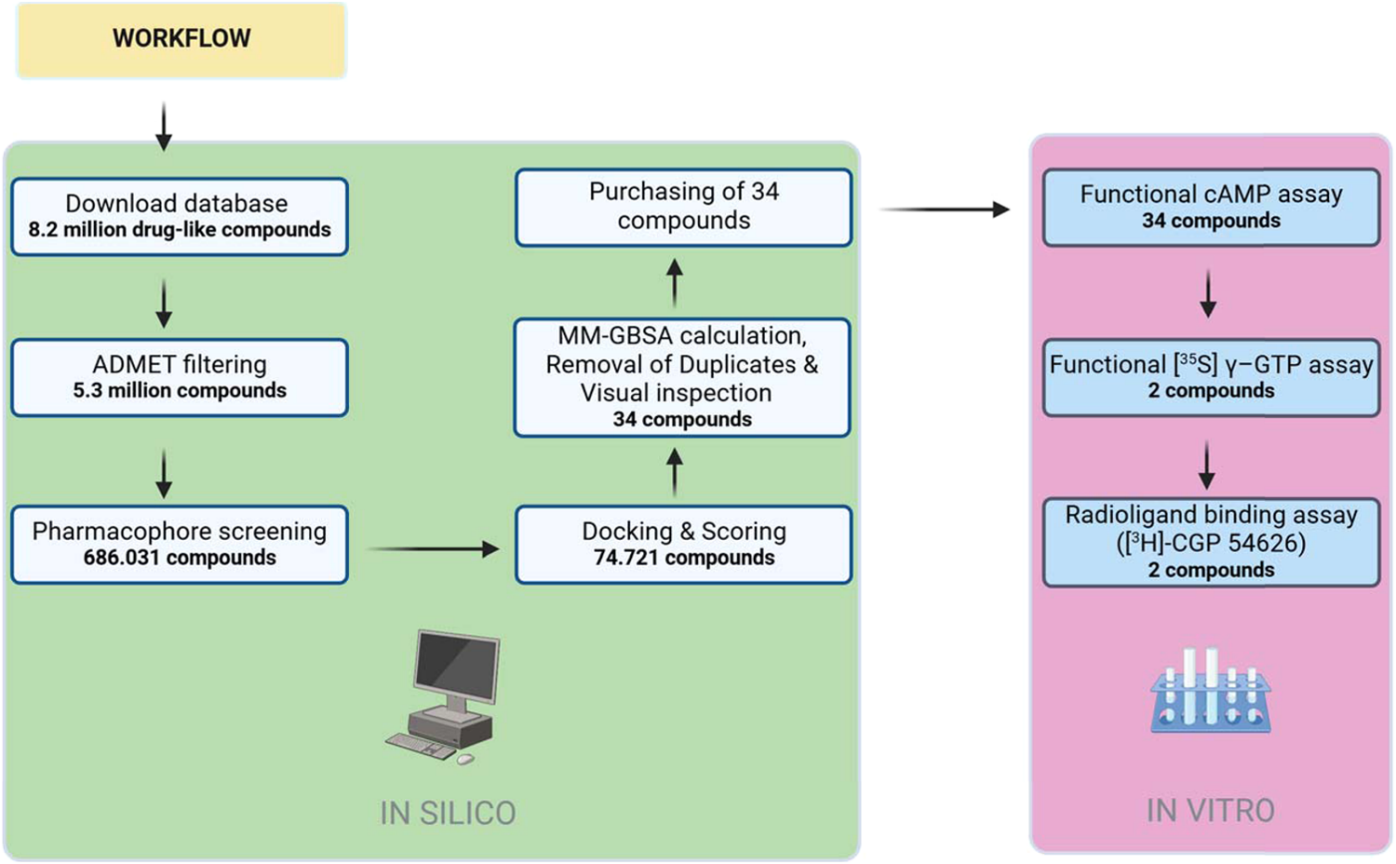
Flowchart summarizing the steps in the *in silico* and *in vitro* workflows. ADMET filtering: absorption, distribution, metabolism, excretion, and toxicity filtering; MM-GBSA: Molecular Mechanics-Generalized-Born Surface Area calculations. Created in BioRender. Russotto, C. (2025), https://BioRender.com/irdtvwe.

In the selection of compounds for experimental testing, we tried to balance the affinity predictions (docking scores and MM-GBSA values) with the number of VFT conformations in which the compounds scored better than thresholds. In addition, we did a visual inspection of the binding patterns to ensure that the compounds interacted with residues previously established as crucial for the binding of agonists and antagonists by Geng and co-workers.^11^ These amino acids included Ser130, Ser153, His170, Glu349, and Trp65 in LB1 and Tyr250 and Trp278 in LB2. The smile codes of the 34 compounds purchased for *in vitro* testing are shown in Table S1.

### Functional Hit Hunter cAMP Assay

The compounds were initially tested using the Hit Hunter cAMP assay.^47^ Activation of the GABA_B_-R leads to G_i/o_ protein coupling, giving inhibition of adenylate cyclase and thus reducing production of the second messenger cAMP. The Hit Hunter cAMP assay measures the cAMP changes, thus giving information about the functional impact on the signaling cascade of the receptor. The 34 compounds were initially tested both in Chinese Hamster Ovary (CHO)-K1 cells overexpressing the GABA_B(1b,2)_-R (GABA_B(1b,2)_-R cells) (Supporting Information, Figure S1) and in wild-type (WT) CHO-K1 cells (Supporting Information, Figure S2). Testing in WT cells was performed to validate that the observed effects on cAMP production in GABA_B(1b,2)_-R cells most probably were caused by GABA_B_-R binding.

The experiments were performed at GABA concentrations of 24.4 and 741 nM, corresponding to EC_20_- and EC_80_-values of GABA. Initial testing indicated that none of the compounds had strong effects on the cAMP production (Figure S1). However, some of the compounds showed relatively weak effects (compounds **2**, **6**, **8**, **16**, **17**, **23**, and **28**) and were therefore tested several times, rendering a trend, as in Figure S1. Based on the initial testing in WT and GABA_B(1b,2)_-R cells, compounds **23** and **28** showing an increase in the cAMP production in GABA_B(1b,2)_-R cells were selected for further testing.

By using a concentration of 10 μM compounds **23** and **28** and varying concentrations of GABA, we tested the effect of the compounds on the dose–response curve of GABA in the Hit Hunter cAMP assay (Figure 2). The curve with GABA only shows a dose-dependent reduction in cAMP production by increasing GABA concentrations. The EC_50_ value for GABA-mediated cAMP reduction was found to be 208 nM ± 44 (±SEM). At low GABA concentrations, both compounds **23** and **28** increased the cAMP levels, indicating a reduction in GABA-mediated receptor stimulation compared with GABA alone (Figure 2). However, at low GABA concentrations, compound **28** increased the cAMP level to a greater extent than compound **23**. ANOVA statistical analysis indicated that 10 μM of compound **28** gave a significant increase in cAMP levels at GABA concentrations up to 10 nM. However, the increase in cAMP levels caused by compound **23** at low GABA concentrations was not found to be significant.

**Figure 2:**
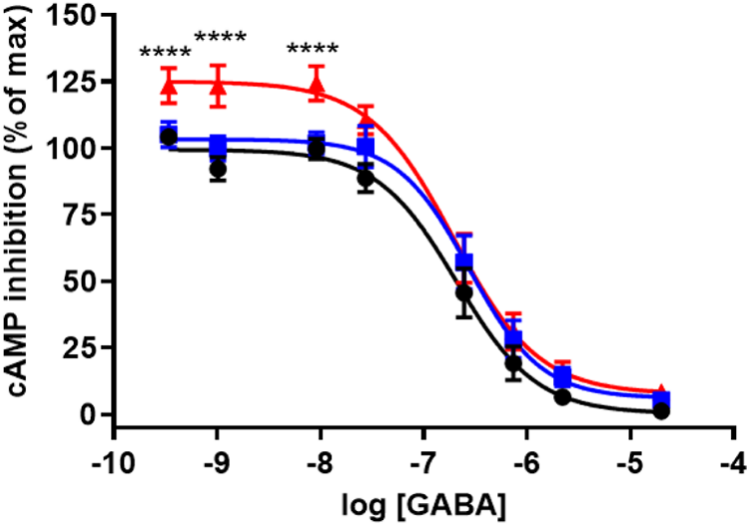
Dose–response effects of compounds **23** (blue squares) and **28** (red triangles) on GABA-induced cAMP inhibition in GABA_B_-R expressing CHO-K1 cells (black circles). Serial dilutions of GABA (20 μM to 0.34 nM) were applied to CHO-K1 cells overexpressing GABA_B(1b,2)_-Rs in the presence or absence of 10 μM compound **23** or **28**. The cAMP levels were measured using the Hit Hunter cAMP assay, where increasing GABA concentrations reduced cAMP production due to GABA_B_-R activation. Data show the shift in GABA’s dose–response curve upon addition of compounds **23** and **28**, highlighting their effects on receptor-mediated cAMP inhibition. Results are expressed as the mean ± SEM of three independent experiments performed in triplicate. Statistical significance of the fit was analyzed by one-way ANOVA. Statistical significance with respect to the GABA-induced cAMP inhibition was analyzed by Welch’s *t* test (**** for *p* < 0.0001).

In the presence of 10 μM compound **23**, the EC_50_ value of GABA increased to 276 nM ± 72 (±SEM), while in the presence of 10 μM **28**, the EC_50_ value slightly decreased to 194 nM ± 56 (±SEM) compared with GABA alone. These results indicate that compound **23** at a concentration of 10 μM may act as a weak antagonist or a NAM on the GABA_B_-R. The results concerning the effects of compound **28** are not conclusive based on the Hit Hunter cAMP assay since we observed a significant reduction in GABA-mediated receptor stimulation at low GABA concentrations, but the EC_50_ value was very similar to that of GABA alone.

### Functional [^35^S]­GTPγS Assay

In the functional [^35^S]­GTPγS assay, G-protein activation is directly measured by tracking the binding of the radiotracer [^35^S]­GTPγS, a nonhydrolyzable analogue of GTP, to the G-protein.^48^ When GABA_B_-R is activated, it facilitates the exchange of GDP for GTP on the α subunit of G-proteins. In this assay, [^35^S]­GTPγS binds to the G-protein in place of GTP, which indicates receptor activation. Therefore, this assay provides a direct measure of the initial activation of G-proteins triggered by receptor-agonist binding, offering insights into the immediate effects of compounds **23** and **28** on GABA-induced G-protein signaling of the GABA_B_-R.

The [^35^S]­GTPγS assay showed that both compounds significantly reduced GABA-induced G-protein activation to approximately 60% at 30 μM concentration after stimulation with 100 μM GABA (Figure 3A). Compound **28** showed a slightly stronger reduction in comparison to compound **23**. This suggests that compound **28** more effectively prevents GABA from promoting G-protein activation.

**Figure 3:**
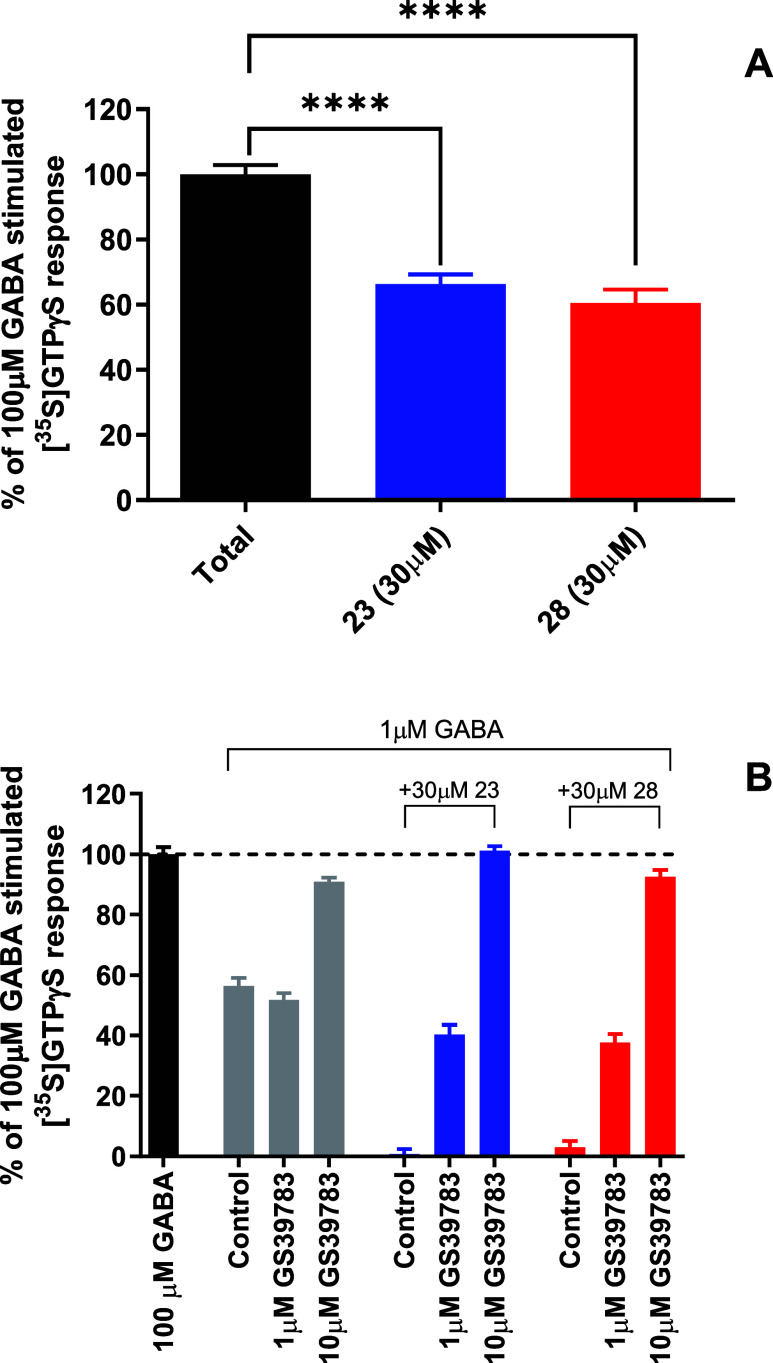
Inhibition of GABA-induced G-protein activation by compounds **23** and **28** in the [^35^S]­GTPγS assay. (A) CHO-K1 cell membranes expressing GABA_B_-Rs were incubated with 30 μM of compounds **23** or **28** in the presence of 100 μM GABA, and [^35^S]­GTPγS binding was measured to assess G-protein activation. (B) CHO-K1 cell membranes expressing GABA_B_-Rs were stimulated by 1 μM GABA only, and in the presence of 1 or 10 μM of PAM GS39783, G-protein activation was monitored. Then, 30 μM of compound **23** or **28** was added, and the G-protein activation was compared with activation without compound **23** or **28** and with the activation of 100 μM GABA. These experiments indicated that both compounds **23** and **28** bind to the orthosteric site. Results are expressed as mean ± SEM of two independent experiments in duplicate. Significant differences were tested by Welch’s *t* test (**** for *p* < 0.001).

To elucidate the intrinsic activity of compounds **23** and **28**, we tested these compounds in the presence of 1 μM GABA with and without two different concentrations of the PAM GS39783 (Figure 3B). The figure shows that 1 μM GABA without test compound and GS39783 gave [^35^S]­GTPγS binding of approximately 55% compared with a concentration of 100 μM GABA. Adding 1 μM PAM did not change the [^35^S]­GTPγS binding, but adding 10 μM increased the binding to almost the same level as for 100 μM of GABA. (Figure 3B). The figure shows that GS39783 potentiates the effect of GABA on [^35^S]­GTPγS binding, indicating that GABA and PAM cooperate to activate the receptor. Adding compound **23** completely abolished the activation caused by 1 μM GABA (control), while compound **28** almost completely abolished this activation. However, adding 1 or 10 μM GS39783 resulted in increased [^35^S]­GTPγS binding, indicating that the PAM potentiated binding of GABA to the receptor despite the presence of compound **23** or **28** and that receptor activation was regained. These results indicate that both compound **23** and compound **28** compete with GABA for the orthosteric binding site of GABA_B_-R and must be weak GABA_B_-R antagonists.

### Competition Binding Assay Using [^3^H]­CGP54626

To further verify that compounds **23** and **28** bind to the orthosteric binding site of the GABA_B_-R, we performed a competition radioligand binding assay using the GABA_B_-R antagonist [^3^H]­CGP54626 as the radioligand (Figure 4). Binding of CGP54626 to the orthosteric site has been confirmed by X-ray crystallographic studies (PDB id: 4MR7). The results showed that both compounds **23** and **28** decreased the specific binding of [^3^H]­CGP54626, indicating that they both bind to the orthosteric site and compete with [^3^H]­CGP54626. Compound **23** decreased the specific binding of [^3^H]­CGP54626 slightly more than compound **28**, indicating that compound **23** displayed receptor occupancy slightly higher than that of compound **28**. Putative binding of [^3^H]­CGP54626 and test compounds to the glass fiber filter used in the experiment was also tested by performing experiments in the absence of cell membranes. Binding of radioligand to the glass fiber filter may interfere with the results and lead to misinterpretation and is an important control in filter-based radioligand binding assays. These experiments showed that the radioligand [^3^H]­CGP54626 in combination with unlabeled CGP54626, **23**, or **28** did not increase the binding of [^3^H]­CGP54626 to the glass fiber filter (Figure S3).

**Figure 4:**
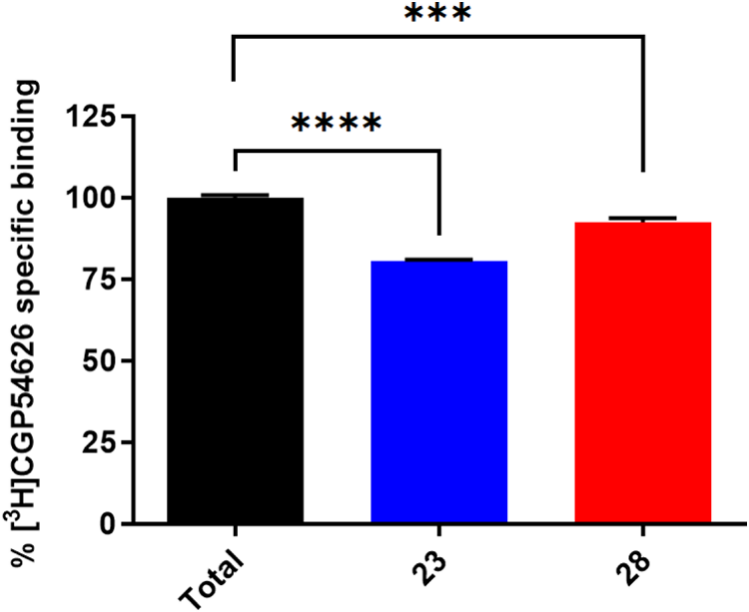
[^3^H]­CGP54626 competition binding assay of compounds **23** and **28** in human GABA_B_-R membranes isolated from Chem-1 cells. The concentration of radioligand in the assay was 5 nM, while the concentration of test compounds **23** and **28** was 20 μM. Values represent mean ± SEM of two independent experiments in duplicate. Significant differences were tested by Welch’s *t* test (*** for *p* < 0.001, **** for *p* < 0.0001).

## Discussion

### Virtual Screening

In VS workflows, knowledge-based computational methods are used to identify new compounds. In general, VS workflows can be broadly categorized into two main approaches: Ligand-Based Drug Discovery (LBDD) methods and Structure-Based Drug Discovery (SBDD) methods.^49^ LBDD methods use information about known compounds (e.g., structure, target affinity/activity, and/or physicochemical properties) to search for new compounds with similar properties. SBDD methods utilize structural information about the drug target to potentially identify new ligands. SBDD methods include methods ranging from docking and scoring to molecular dynamics-based methods to estimate the binding energy.^50,51^ In our previous study, we evaluated different LBDD and SBDD methods for their suitability to identify orthosteric GABA_B_-R compounds.^34^ Based on this study, we selected a combination of appropriate LBDD and SBDD methods for the present VS workflow. The selected methods were used to screen databases of altogether 8.2 million compounds from different vendors, aiming to identify new compounds binding to the orthosteric binding site of GABA_B_-R.

One reason for the higher number of compounds scoring better than the threshold value by docking into the antagonist-based VFT structures may be that they are in a more open and accessible conformation than the agonist-based VFT conformation. In addition, we had six representations of the VFT complexed with antagonists but only two representations of the VFT complexed with agonists, which contributes to a broader conformational space of the VFTs representing the open inactive conformation than the closed active conformation. The compounds after docking were further evaluated by Molecular Mechanics-Generalized-Born Surface Area (MM-GBSA) calculations, which is a more accurate estimate of the binding energy than docking scores.^50^

### *In Vitro* Evaluation

Based on the *in vitro* experiments, we can conclude that both compound **23** and compound **28** occupy the orthosteric binding site of GABA_B_-R and are weak GABA_B_-R antagonists. GABA dose–response curves (Figure 2) showed that 10 μM compound **28** significantly reduced GABA-induced inhibition of cAMP production at low GABA concentrations (up to 10 nM), while 10 μM compound **23** showed nonsignificant effects at low GABA concentrations. Although not significantly different with respect to GABA alone or GABA together with compound **28**, compound **23** showed a stronger overall effect on the GABA-induced inhibition of cAMP compared to compound **28**. This was demonstrated by the EC_50_ values that increased from 208 nM for GABA alone to 276 nM for GABA in combination with compound **23**. The EC_50_ value for GABA in combination with compound **28** was 194 nM. However, the [^35^S]­GTPγS assay showed that at a concentration of 30 μM compound **28** reduced the response of 100 μM GABA slightly more than compound **23** (Figure 3). cAMP is a measure of downstream signaling, while the higher efficacy observed in the [^35^S]­GTPγS assay for compound **28** suggests that it has a stronger antagonizing action on GABA signaling at the G-protein level than compound **23**, while compound **23** is a stronger antagonist of the cAMP pathway at high GABA concentrations than compound **28**.

In the competition binding assay, compound **23** decreased the specific binding of the antagonist [^3^H]­CGP54626 more potently than compound **28** (Figure 4). The observation that compound **23** displays slightly higher receptor occupancy but slightly weaker antagonism than compound **28** aligns with findings in previous GPCR research, where the receptor binding affinity (occupancy) of a ligand may not directly correlate with its functional efficacy. This discrepancy can occur because some ligands stabilize receptor conformations that do not effectively promote downstream signaling, thus occupying the receptor without fully activating or antagonizing the receptor.^52,53^ Essentially, while a weak antagonist like compound **23** may bind well to the GABA_B_-R orthosteric site, it might not stabilize a conformation that robustly antagonizes receptor activation, resulting in a slightly weaker functional response compared to compound **28**. This phenomenon is common in GPCR pharmacology, where the specific conformational state stabilized by a ligand can determine its efficacy even if the ligand binds the receptor with low affinity. This concept, called binding efficacy separation, highlights that occupancy and efficacy can diverge, particularly for antagonists that may block receptor activity without inducing a strong conformational shift.^52^

Using the competition binding assay, we also tried to obtain IC_50_/*K_i_* values of compounds **23** and **28**. These compounds are weak antagonists, such that very high concentrations of the compounds are required, and IC_50_/*K_i_* values of compounds **23** and **28** for the GABA_B_-R will therefore be very uncertain. In these experiments, we were using a concentration of 4 nM of [^3^H]­CGP54626, while compounds **23** and **28** were diluted six times in the concentration range of 123 nM to 100 μM. For compound **23**, this resulted in an IC_50_ value of 23.3 μM (n = 1), while we were not able to obtain a decent dose–response curve in this concentration range for estimating the IC_50_ value of compound **28**. In order to test the performance of our system, we also estimated the IC_50_/*K_i_* values of CGP54626 using six dilutions and a concentration of 4 nm of [^3^H]­CGP54626. The obtained IC_50_ value was 20 nM, giving a *K_i_* value of 4.5 nM. Hirst and co-workers obtained a *K_i_* value of 7.08 nM using another membrane system than we are using,^54^ indicating that our system is well functioning. Compound **28** is a weaker antagonist than compound **23** (Figure 4), but compound **28** also has some structural similarities to toxoflavin, which is a toxin produced by different bacteria. Toxoflavin is known to be a Pan-assay Interference compound that may cause problems in biological assays.^55^ Our results show that compound **28** binds to the orthosteric site of the GABA_B_-R (Figures 2–4), but it may be possible that the off-target unspecific binding is high at very high concentrations of the compound, causing problems in the tested concentrations’ range. However, the compound was not filtered out by the ADMET filters during VS that were used to retrieve drug-like compounds.

Both compounds **23** and **28** were retrieved from agonist-based pharmacophore models during the ligand-based steps of the VS process (Figure 1), but the *in vitro* results showed that they are antagonists. However, most known antagonists are based on the structure of GABA, and previous studies have shown that the structural differences between the GABA_B_-R agonists and antagonists are small. For example, the clinical drug (R)-baclofen is an agonist, while the structural analogues, (R)-phaclofen and (S)–OH-saclofen, are weak antagonists.^11^ As shown by the eight available X-ray structures of GABA_B_-VFTs, the structural similarities between agonists and antagonists are also reflected in their receptor binding modes. Key amino acids for ligand binding are very similar between the six cocrystallized antagonists and the two cocrystallized agonists, GABA and (R)-baclofen.^11,56^ In addition, the root-mean square deviation (RMSD) between the VFTs also indicates structural similarities.^11^ The VFT X-ray structures showed that both agonists and the six antagonists interact with Ser130, Ser153, His170, Glu349, and Trp65 in LB1, while the agonists and the two antagonists, SCH509 and CGP54626, are also directly engaged in binding to amino acids in LB2 (Tyr250 and Trp278). The binding site is more open in the antagonist-based VFTs and should adopt compounds larger than those in the agonist-based VFTs. However, the similarities in key amino acids for binding between agonists and antagonists caused the generation of highly similar grid maps for docking into the different VFTs, and thereby, there is the possibility that the structure-based steps in the present study would retrieve compounds with limited structural diversity. Based on this, we decided to study the binding modes of compounds **23** and **28** with the orthosteric site of the GABA_B_-R and their structural similarities with known GABA_B_-R compounds.

To compare the binding mode of compounds **23** and **28** with known GABA_B_-R compounds, induced fit docking experiments were performed using an X-ray structure representing an inactive receptor conformation complexed with the antagonist CGP54626 (PDB id: 4MR7). [^3^H]­CGP54626 was used in our competition binding assay. As previously explained, key amino acids for agonist and antagonist binding are very similar between the eight available X-ray structures of VFTs.^11,56^ During the induced fit docking process, amino acids within five of the ligands were optimized, giving the possibility of inducing other conformations of the orthosteric binding site. The induced fit docking experiments indicated that the VFT interaction patterns of these compounds were highly like those of known agonists and antagonists.^11,56^ As seen in Figure 5, they all formed hydrogen bonds to key amino acids in LB1 previously found to be important in the binding of known agonists and antagonists. Similarly to CGP54626, both compounds **23** and **28** interacted with Trp278 in LB2. After induced fit docking, the binding score for compound **23** was −8.4 kcal/mol, while the score for compound **28** was −3.7 kcal/mol. That compound **23** scored better than compound **28** agrees with the results from the competition binding assay (Figure 4). The scoring value for compound **28** was not better than the threshold value for antagonist-based VFTs (−7.1 kcal/mol), but this compound was retrieved by an agonist-based pharmacophore model and was docked into agonist-based and not antagonist-based VFTs during the VS.

**Figure 5:**
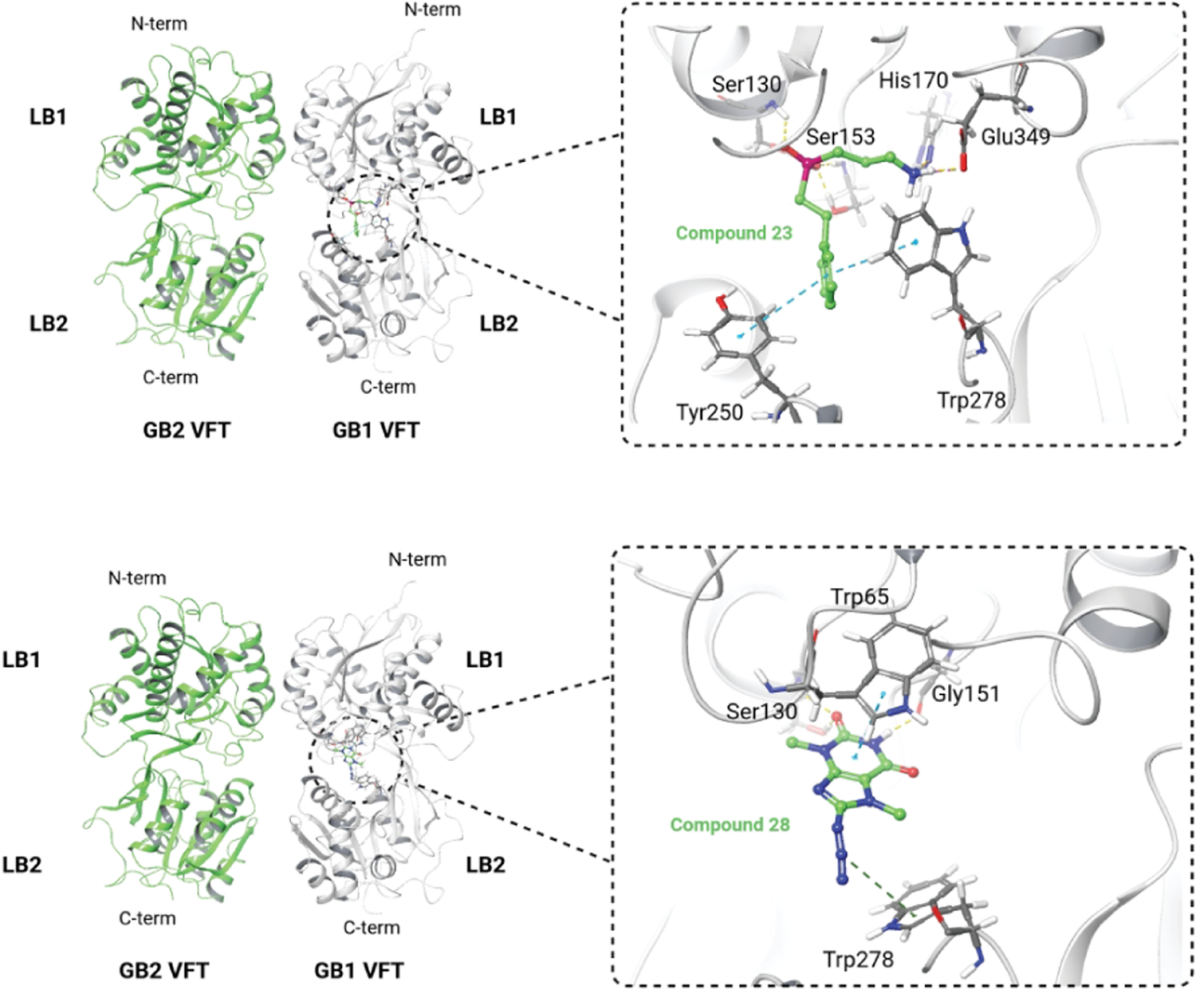
Induced fit docking of compound **23** and compound **28** into the X-ray structure of GABA_B1_-R VFT (PDB id: 4MR7). Color coding of atoms in compounds **23** and **28**: nitrogen, blue; oxygen, red; phosphor, dark red; carbon, green; hydrogen, white.

The structural similarities of compounds **23** and **28** with 177 known GABA_B_-R compounds were evaluated using hierarchical clustering. We created a new data set consisting of GABA_B_-R agonists, -antagonists, -PAMs, and -NAMs. The clustering procedure divided the data set into 15 clusters using the Kelly criterion^42^ and 0.9164 metric distance to select the optimal number of clusters. The number of compounds in each cluster is shown in Table 1. Compound **23** was in cluster 5, consisting of 11 known antagonists and two agonists (Table 1). Structures are usually considered similar if the Tanimoto similarity coefficient (*T*_c_) is >0.85.^46^ The compounds in cluster 5 with the highest structural similarities with compound **23** were the antagonist CGP36742 (*T*_c_ = 0.437), the antagonist CGP46381 (*T*_c_ = 0.437), and the agonist 3-APMA (*T*_c_ = 0.428). The Tanimoto similarity coefficients indicated that compound **23** has quite large structural similarities with known GABA_B_-R compounds (Figure 6). However, compound **28** was the only compound in cluster 10 (Table 1). Compounds with the largest structural similarity with compound **28** were the putative NAM 13c (*T*_c_ = 0.066) from cluster 11, the antagonist CGP71872 (*T*_c_ = 0.039) from cluster 5, and the antagonist CHEMBL123039 (*T*_c_ = 0.033) from cluster 13. This indicates that compound **28** has a unique structure compared with the presently known GABA_B_-R agonists and antagonists.

**Figure 6:**
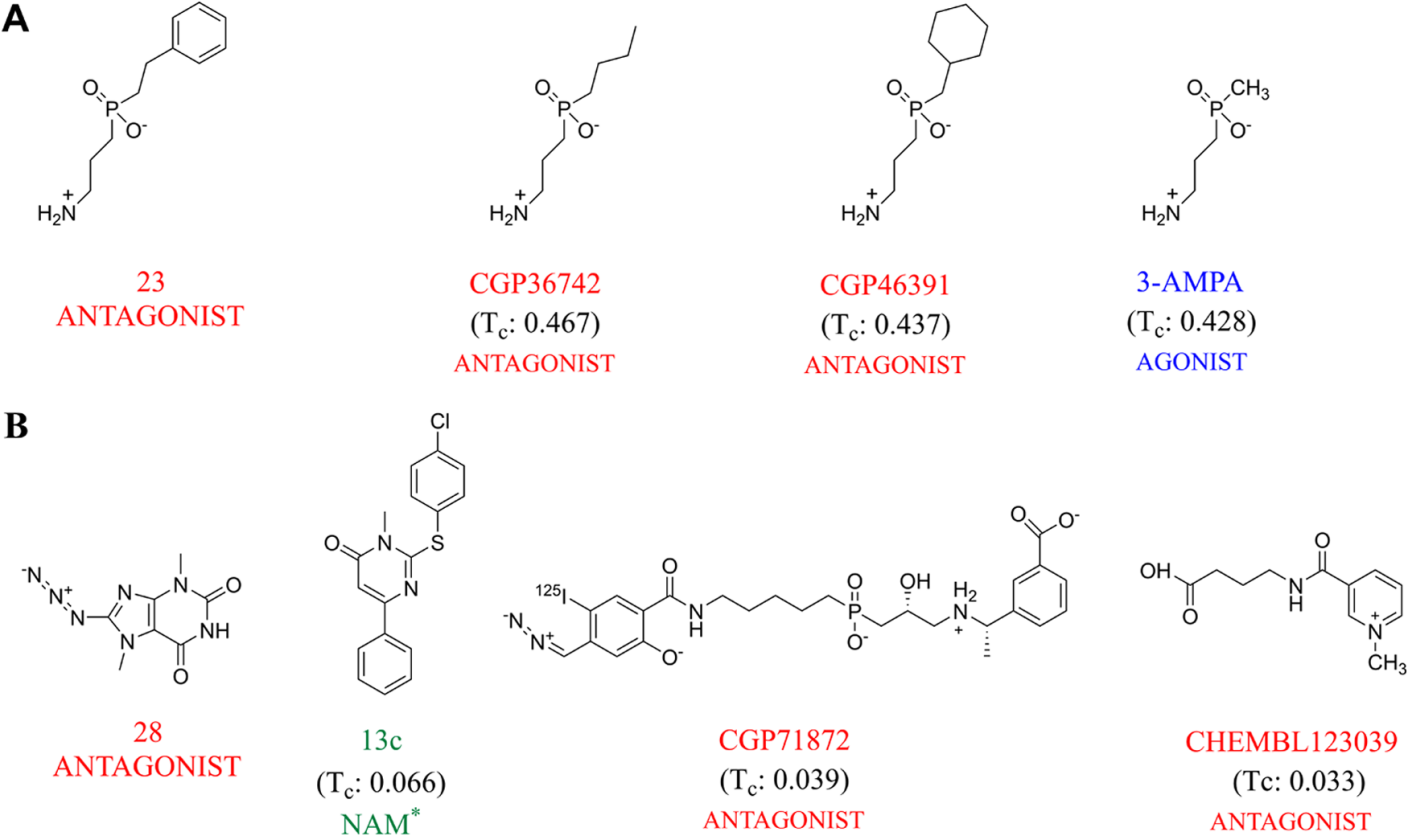
Structure, functional receptor activity, and Tanimoto similarity coefficient (*T*_c_) of known GABA_B_-R compounds with the largest structural similarity to compound **23** (A) and **28** (B). *13c is a putative NAM.^44^

**Table 1:** Hierarchical Clustering of Compounds **23** and **28** with Known GABA_B_-R Compounds

cluster	number of compounds (including tautomers)	activity
1	16	1 agonist, 2 PAMs, 8 NAMs
2	44	43 PAMs
3	1	1 NAM
4	32	14 agonists
5	18	11 antagonists, 2 agonists, compound **23**
6	1	1 NAM (COR758)
7	1	1 agonist
8	1	1 agonist
9	39	19 agonists, 2 antagonists
10	1	Compound **28**
11	1	1 NAM (13c)
12	1	1 antagonist
13	7	3 antagonists
14	14	1 antagonist, 6 agonists
15	2	2 agonists

## Conclusions

A combination of ligand-based and structure-based VS was used to screen a library of 8.2 million compounds to identify new ligands for GABA_B_-R. After the VS, 34 compounds were purchased for *in vitro* studies. All 34 compounds were initially tested by using the functional Hit Hunter cAMP assay in CHO-K1 cells overexpressing GABA_B(1b,2)_-R and in WT CHO-K1 cells. Based on the initial testing, two compounds (compounds **23** and **28**) that increased cAMP production were selected and tested for their effects on the dose–response curve of GABA using the Hit Hunter cAMP assay. These experiments showed that compound **28** gave a significant antagonistic effect at low GABA concentrations (<10 nM), while compound **23** gave a higher increase of the GABA EC_50_ value than compound **28**, indicating that compound **23** is a better antagonist of the GABA_B_-R cAMP pathway than compound **28**. Based on the Hit Hunter assay, we could conclude that compound **23** is a weak GABA_B_-R antagonist or an NAM, while the assay was not conclusive for compound **28**. Testing the compound in the [^35^S]­GTPγS assay indicated that both compounds bind to the orthosteric site of the receptor and significantly reduce GABA-induced G-protein activation to approximately 60% at 30 μM concentration after stimulation with 100 μM GABA. Compound **28** showed a slightly stronger reduction in activation compared to compound **23**, suggesting that compound **28** is more effective in preventing GABA from activating G-proteins. Therefore, the [^35^S]­GTPγS assay indicated that both compounds are weak GABA_B_-R antagonists. Further, the [^3^H]­CGP54626 radioligand binding assay showed that both compounds compete with GABA for an orthosteric binding site. Compound **23** had slightly higher receptor occupancy than compound **28**.

Similarities in docking mode with known GABA_B_-R agonists and antagonists were one of the criteria for selecting compounds for *in vitro* evaluation. The available VFT X-ray structures show that only the two antagonists SCH509 and CGP54626 and agonists GABA and (R)-baclofen interact with amino acids in both LB1 and LB2, while all other cocrystallized compounds are antagonists and only interact with amino acids in LB1. CGP54626 has a high binding affinity for the GABA_B_-R and interacts with amino acids both in LB1 and in LB2.^11^ Induced fit docking indicated that compounds **23** and **28** interact with Trp278 in LB2 but not with other amino acids in LB2. A stronger focus in the selection process of compounds for *in vitro* testing on compounds with stronger interactions with amino acids in LB2 may have given compounds with a stronger receptor affinity.

Our experimental studies show that both compounds are weak GABA_B_-R antagonists. GABA is the main inhibitory neurotransmitter of the CNS, and antagonism of GABA_B_-R by compounds with high affinity and efficacy may therefore result in a broad range of side effects as seen for the agonist baclofen. An overdose of baclofen may be potentially fatal.^57^ Antagonists or agonists with quite low affinity and efficacy that fine-tune the receptor activity instead of completely activating or antagonizing the receptor may therefore be interesting for therapeutic interventions if the pharmacokinetic properties are satisfactory. Compounds **23** and **28** in the present study may therefore be used as starting points for developing new drugs targeting the GABA_B_-R.

## Supplementary Material









## Data Availability

The data associated with this work are provided with the paper or as Supporting Information. Compound structures for VS were downloaded from the ZINC 15 database (http://zinc15.docking.org). X-ray structures for VS (PDB id: 4MS3, 4MS4, 4MR7, 4MR8, 4MR9, 4MS1, 4MRM, 4MQF) and induced fit docking (PDB id: 4MR7) were downloaded from the Protein Data Bank (PDB: http://www.rcsb.org/). Compounds for similarity testing were downloaded from ChEMBL (https://www.ebi.ac.uk/chembl/) and IUPHAR (https://www.guidetopharmacology.org/). The 34 compounds for *in vitro* testing were purchased from Molport (https://www.molport.com). Ligand- and structure-based VS, hierarchical clustering and induced fit docking were performed with different versions of the Schrödinger suite of programs (http://www.schrodinger.com) distributed under licenses. Data-handling, curve fitting, and statistical analysis of *in vitro* results were performed with the licensed software GraphPad Prism v. 9.0 (https://www.graphpad.com/updates/prism). Figure 1 was generated by the licensed software BioRender (https://www.biorender.com).

## Notes

Compounds selected for *in vitro* testing. Smiles code, Molport id, zinc 15 id, predicted functional activity, and pharmacophore model retriving the compound are given (Table S1); results of the initial screening of the 34 compounds in CHO-K1 cells stably expressing human GABA_B(1b,2)_ receptors using the Hit Hunter cAMP assay (Figure S1); results of screening of the 34 compounds in wild-type CHO-K1 cells using the Hit Hunter cAMP assay (Figure S2); results of experiments showing that compounds **23** and **28** did not increase the binding of [^3^H]­CGP54626 to the filter paper (Figure S3) (PDF)
“*From pharmacophores-clustering of known compounds*” with the following compound information: The sheet named “*From agonist pharmacophores*” contains compounds retrieved by agonist-based pharmacophores (including cluster 1), while the sheet named “*From antagonist pharmacophores*” contains compounds retrieved by the antagonist-based pharmacophores (including cluster 1) that were included in the final selection of compounds for in vitro testing, the two sheets contain their ZINC 15 identification number, XP docking score (gscore), MM-GBSA energy, and the number of VFTs where they scored better than the threshold value. The sheet named “*Clustering_GABA-B_compounds*” contains the GABAB-R compounds used for hierarchical clustering (see Table 1); Smiles code, molecular identification number, intrinsic activity, and cluster number of each compound are given (XLSX)
Complexes in PDB-format of compounds **23** and **28** after induced fit docking into the GABAB-VFT (PDB id: 4MR7) (PDB, PDB)
