# Supplementary material for: Identification of Orthosteric GABAB Receptor Ligands by Virtual Screening and In Vitro Validation
Source: ACS Omega. 2025 May 16;10(21):22005–17. doi: 10.1021/acsomega.5c02102 (PMC12138688; doi:10.1021/acsomega.5c02102)
Supplement: Supplementary file 1 [file ao5c02102_si_001.pdf]

Supporting information

Identification of Orthosteric $GABA_B$ Receptor Ligands by Virtual Screening and *in vitro* Validation

Linn S. M. Evenseth[1]‡, Clizia Russotto[1]‡, Imin Wushur[1], Dawid Warszycki[2], Angel S. Moldes-Anaya[3], Andrzej J. Bojarski[2], Mari Gabrielsen[1] and Ingebrigt Sylte[1,4,*]

[1]Pharmacology and Toxicology, Department of Medical Biology, Faculty of Health Sciences, UiT – The Arctic University of Norway, NO-9037, Tromsø, Norway.
[2]Department of Medicinal Chemistry, Maj Institute of Pharmacology, Polish Academy of Science, Kraków, Poland.
[3]Cyclotron and Radiochemistry Unit, Section for Radiopharmaceutical Production, The PET Imaging Center, University Hospital of North Norway (UNN), Tromsø, Norway.
[4]Center for Research and Education, University Hospital of North Norway (UNN).

‡: These authors have contributed equally to the work.

*: Corresponding author.

In the present file:

Table S1. Compounds selected for *in vitro* testing, page S3.

Figure S1. Screening in CHO-K1 cells stably expressing human $GABA_{B(1b,2)}$ receptors using the Hit hunter cAMP assay, page S4.

Figure S2. Screening in wild type CHO-K1 cells using the Hit hunter cAMP assay, page S5.

Figure S3. Experimental tests to check if compound 23 and 28 increase filter paper binding of [^{3}H]CGP54626, page S6.

Table S1. Compounds selected for *in vitro* testing. Compound number in the present study, smiles code, molport id, zinc15 id, predicted functional activity, and the pharmacophore model (cluster) retrieving the compound during pharmacophore screening are given.

Compound number	Smiles code	Molport id	Zinc15 id	Predicted activity	Cluster
1	CC(Sc1nnc(c2c[nH]c3ccccc23)n1C1CC1)C(=O)N1CCNC1=O	004-156-972	ZINC07872819	Antagonist	C1
2	O=c1[nH]c(CN(CCc2ccccc2)Cc2ccccc2)nc2ccsc12	004-224-257	ZINC57315282	Antagonist	C3
3	CN(Cc1nc2ccsc2c(=O)[nH]1)Cc1ccc(Cl)cc1Cl	005-525-376	ZINC23731134	Antagonist	C3
4	CC(Sc1nnc(C2COc3ccccc3O2)n1C)C(=O)N1CCNC1=O	005-536-313	ZINC08938299	Antagonist	C1
5	CN(Cc1ccc(Cl)c(Cl)c1)Cc1nc2ccsc2c(=O)[nH]1	005-540-667	ZINC14018329	Antagonists	C3
6	CN(CCOc1ccccc1Cl)Cc1nc2ccsc2c(=O)[nH]1	005-542-252	ZINC12679880	Antagonist	C3
7	CC(N(C)Cc1nc2ccsc2c(=O)[nH]1)c1cc(F)ccc1F	009-081-084	ZINC53125897	Antagonist	C3
8	CCN(Cc1ccc(F)c(F)c1)Cc1nc2ccsc2c(=O)[nH]1	009-081-338	ZINC53209882	Antagonist	C3
9	Cl.COC(=O)CC(N)c1ccc(Br)cc1	009-141-584	ZINC00170062	Agonist	C6
10	CCCCNc1nnc(SC(C)C(=O)N2CCNC2=O)s1	009-388-475	ZINC32774430	Antagonist	C1
11	Cl.Cl.NC(Cn1ccnc1)c1cccc(c1)C(F)(F)F	009-473-776	ZINC22565256	Agonist	C6
12	NCCC(O)(c1ccccc1)C(F)(F)F	016-635-565	ZINC57218406	Agonist	C5
13	Cl.COC(=O)C(CN)Cc1cccc(F)c1	016-636-403	ZINC20579368	Agonist	C5
14	Cl.COC(=O)CCC(N)c1ccccc1	016-636-410	ZINC36379899	Agonist	C6
15	Cl.COC(=O)C(CN)CC1CCOCC1	016-636-647	ZINC52175727	Agonist	C6
16	CC1CN(C(=O)CCN2CCSC2=O)C(S1)=Nc1ccc(Cl)c(Cl)c1 |w:16.18|	044-263-356	ZINC53125897	Antagonist	C3
17	NNc1nc2ccccc2nc1O	000-249-003	ZINC08951611	Agonist	C6
18	Clc1ccc(NC(=O)CSc2nc(=O)[nH]c3CCCCc23)cc1Cl	007-889-865	ZINC04317076	Antagonist	C1
19	Cc1ncc(CSCC(=O)Nc2cc(cc(c2)C(F)(F)F)C(F)(F)F)c(CO)c1O	002-889-755	ZINC01033175	Antagonist	C1
20	CC1=C(C(NC(=O)N1)c1ccccc1Cl)C(=O)OCCSc1ccccc1 |t:1|	000-908-456	ZINC04066600	Antagonist	C1
21	COc1cc(Br)c(cc1OC)C1CC(=O)NC(C)=C1C(=O)OCc1ccc(C)cc1 |c:18|	000-915-254	ZINC09559861	Antagonist	C1
22	COC(=O)CC(CN)c1ccc(C)cc1	002-566-790	ZINC04811770	Agonist	C5
23	NCCCP(O)(=O)CCc1ccccc1	002-570-298	ZINC20029233	Agonist	C4
24	COc1ccc2C(CN)OC(=O)c2c1OC	006-830-317	ZINC11919650	Agonist	C5
25	CSCCC(NC(=O)N1CC(O)=Nc2ccccc12)C(=O)NCc1ccc(Cl)cc1Cl |c:11|	000-845-295	ZINC13551964	Antagonist	C1
26	CNCC(O)Cn1ccc2ccccc12	002-018-032	ZINC00818950	Agonist	C6
27	CNCC(O)c1c[nH]c2ccccc12	002-343-267	ZINC02008722	Agonist	C6
28	Cn1c(N=[N+]=[N-])nc2n(C)c(=O)[nH]c(=O)c12	002-546-060	ZINC13756475	Agonist	C2
29	COc1cc(ccc1O)C(CN)CC(O)=O	002-564-702	ZINC04856440	Agonist	C4
30	Cc1cc(C(O)=O)c(C#N)c(O)n1	002-913-206	ZINC20458562	Agonist	C2
31	COC(=O)CC(N)c1ccc(Cl)cc1Cl	002-932-567	ZINC04602000	Agonist	C5
32	COC(=O)CC(N)c1ccc(Cl)c(Cl)c1	002-955-003	ZINC04298871	Agonist	C6
33	CC(N)CNS(=O)(=O)c1ccc(Cl)cc1	002-988-817	ZINC09426200	Agonist	C5
34	NCCc1c([nH][nH]c1=O)C1CC1	008-326-849	ZINC12404877	Agonist	C6

Figure S1

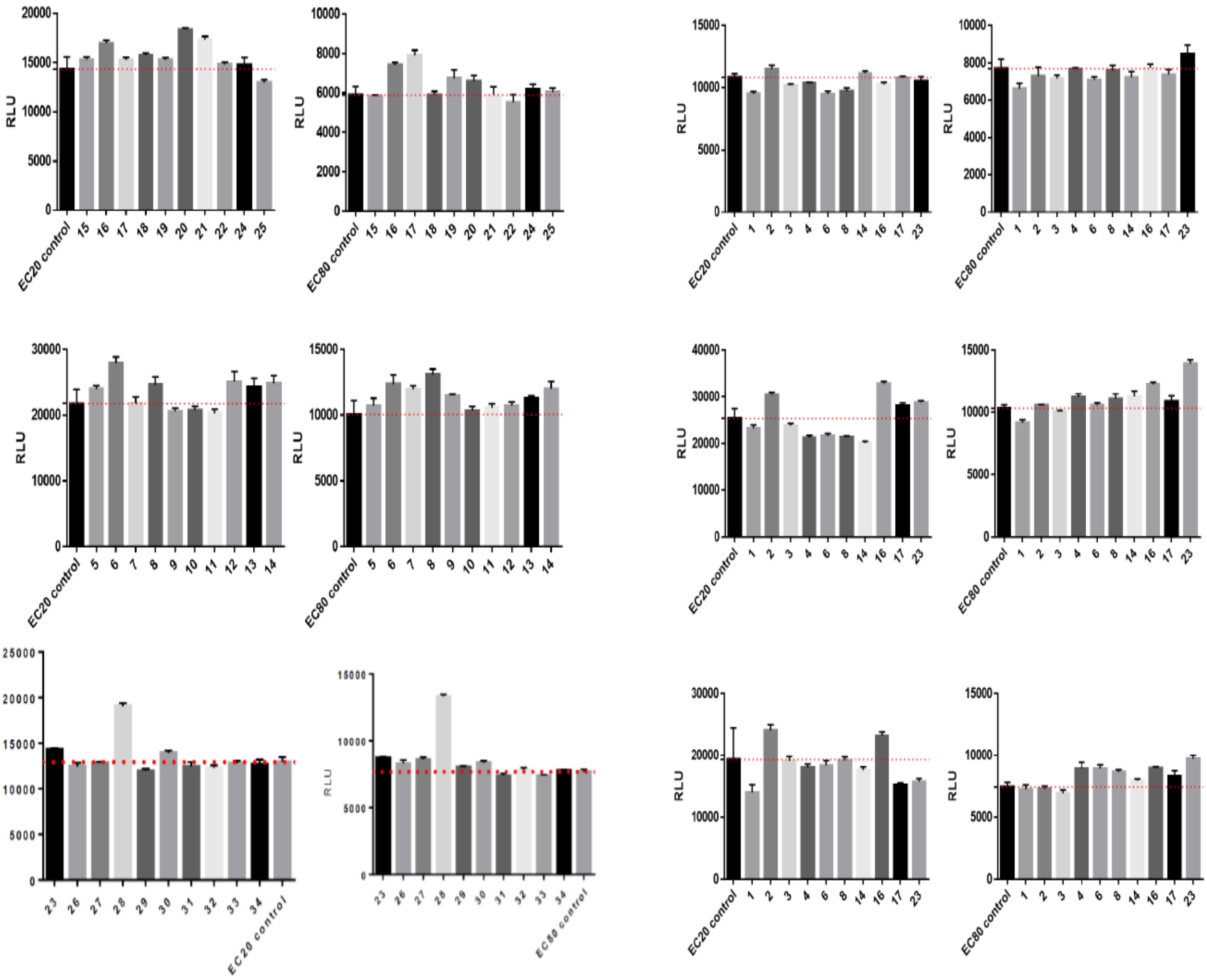

Figure S1. Screening in CHO-K1 cells stably expressing human GABA$_{B(1b,2)}$ receptors using the Hit hunter cAMP assay. The screening was performed as described in the material and methods section at EC$_{20}$ and EC$_{80}$ concentrations of GABA. A concentration of 10 μM was used for the test compounds, except for compound 5 and 25 that were tested in 5 μM concentrations due to solubility problems.

Figure S2

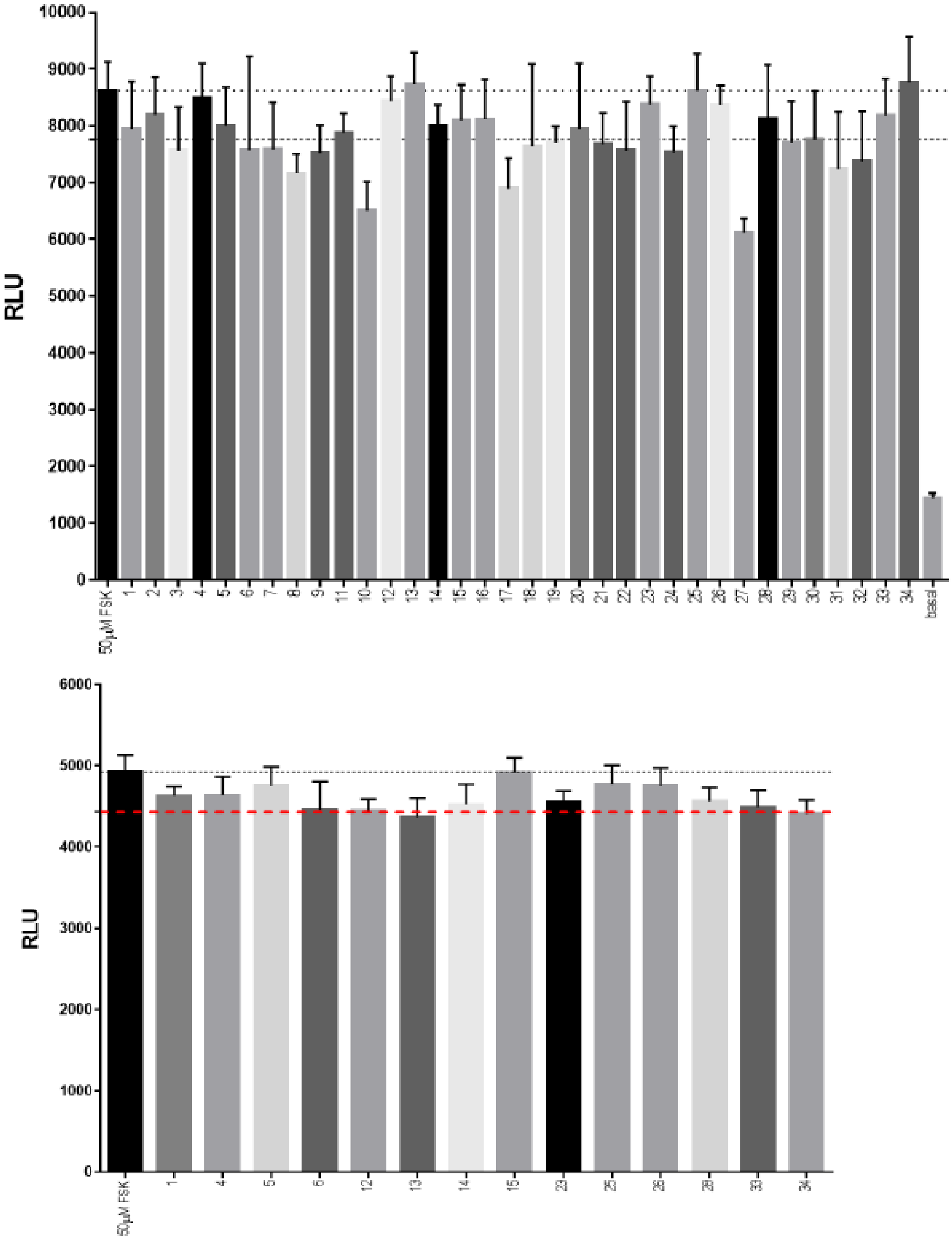

Figure S2. Screening in wild type CHO-K1 cells using the Hit hunter cAMP assay. The screening was performed as described in the material and methods section. A concentration of 10 µM was used for the test compounds, except for compound 5 and 25 that were tested in 5 µM concentrations due to solubility problems.

Figure S3

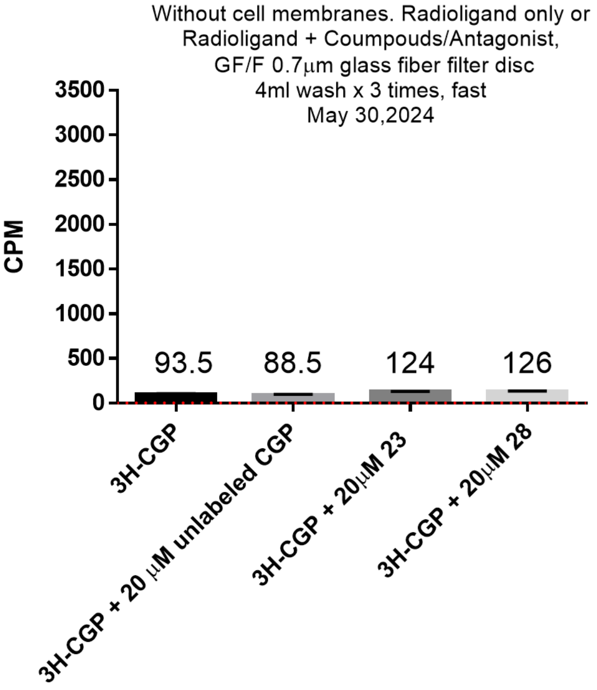

Figure S3. Experiments showing that compounds 23 and 28 not increase binding of [^{3}H]CGP54626 to the filter paper. The experiments were performed without cell membrane preparations.
